# Supplementary material for: Anxious Brains: A Combined Data Fusion Machine Learning Approach to Predict Trait Anxiety from Morphometric Features
Source: Sensors (Basel). 2023 Jan 5;23(2):610. doi: 10.3390/s23020610 (PMC9863274; doi:10.3390/s23020610)
Supplement: Supplementary file 1 [file sensors-23-00610-s001.zip › Table S1.pdf]

Supplementary Table S1. Brain areas for Network 3 (IC-GM 4/IC-WM 1), according to the Talairach daemon

Independent Component IC-GM 4 Positive Values			
**Area**	**Brodmann Area**	**Volume (cc)**	**MNI (x, y, z)**
Fusiform Gyrus	18, 19, 20, 36, 37	5.0/4.5	(-54, -33, -28)/(55, -31, -28)
Inferior Temporal Gyrus	20, 21, 37	4.0/4.6	(-45, -19, -34)/(48, -21, -34)
Parahippocampal Gyrus	20, 35, 36	1.3/1.6	(-39, -25, -25)/(42, -30, -28)
Sub-Gyral	20	1.7/2.4	(-45, -27, -25)/(42, -27, -25)
Uncus	20, 28	0.4/1.2	(-39, -13, -34)/(39, -13, -33)
Culmen	*	1.0/0.9	(-43, -37, -31)/(37, -31, -30)
Middle Temporal Gyrus	20, 21, 37	0.8/3.7	(-55, -40, -19)/(58, -39, -19)
Declive	*	2.4/0.3	(-12, -84, -27)/(31, -84, 30)
Inferior Occipital Gyrus	17, 18	2.0/0.1	(-28, -87, -16)/(24, -88, -21)
Lingual Gyrus	17, 18	2.1/0.4	(-13, -90, -21)/(10, -87, -19)
Middle Occipital Gyrus	18	1.5/0.0	(-28, -84, -13)/(0, 0, 0)
Insula	13	1.1/0.0	(-37, -28, 18)/(0, 0, 0)

Independent Component IC-WM 1 Positive Values			
**Area**	**Brodmann Area**	**Volume (cc)**	**MNI (x, y, z)**
Middle Frontal Gyrus	6, 8, 9	3.3/2.0	(-30, 18, 46)/(37, 13, 43)
Precentral Gyrus	6, 9, 44	0.4/1.1	(-33, 21, 40)/(34, 13, 40)
Inferior Occipital Gyrus	17, 18, 19	0.0/1.4	(0, 0, 0)/(28, -85, -10)
Sub-Gyral	*	2.8/3.9	(-24, 27, 37)/(28, -85, -6)
Lingual Gyrus	17, 18	0.0/2.9	(0, 0, 0)/(25, -88, -10)
Middle Occipital Gyrus	18, 19	0.0/4.6	(0, 0, 0)/(25, -85, 13)
Inferior Parietal Lobule	40	0.0/4.8	(0, 0, 0)/(51, -36, 31)
Cuneus	17, 18	0.0/3.1	(0, 0, 0)/(24, -84, 9)
Fusiform Gyrus	20, 36	1.1/1.2	(-39, -27, -30)/(49, -36, -27)

Independent Component IC-WM 1 Negative Values			
**Area**	**Brodmann Area**	**Volume (cc)**	**MNI (x, y, z)**
Sub-Gyral	*	11.5/13.9	(-39, -52, 10)/(31, -55, 37)
Inferior Parietal Lobule	7, 40	0.0/1.2	(0, 0, 0)/(31, -55, 43)
Superior Temporal Gyrus	21, 22, 39	3.5/2.1	(-42, -55, 10)/(37, -60, 28)
Cuneus	7, 18, 19	0.0/1.9	(0, 0, 0)/(21, -76, 28)
Middle Temporal Gyrus	19, 21, 22, 39	6.4/0.6	(-45, -58, 10)/(34, -63, 28)
Precuneus	7, 19, 31	0.0/4.9	(0, 0, 0)/(25, -58, 40)
Middle Frontal Gyrus	6, 9	0.3/1.4	(-37, -1, 45)/(37, 21, 31)
Extra-Nuclear	13	2.9/3.5	(-31, 9, -4)/(33, 7, -9)
Inferior Frontal Gyrus	13, 47	1.3/0.3	(-37, 33, 6)/(36, 34, 3)
Insula	13, 22	0.4/1.0	(-31, 15, -4)/(31, 13, -3)
Tuber	*	0.4/1.0	(-37, -58, -36)/(37, -58, -36)
Pyramis	*	0.7/1.2	(-15, -67, -37)/(18, -66, -36)
Culmen	*	0.2/1.0	(-37, -55, -39)/(34, -61, -34)
