# Supplementary figures and images for: Anxious Brains: A Combined Data Fusion Machine Learning Approach to Predict Trait Anxiety from Morphometric Features
Source: Sensors (Basel). 2023 Jan 5;23(2):610. doi: 10.3390/s23020610 (PMC9863274; doi:10.3390/s23020610)

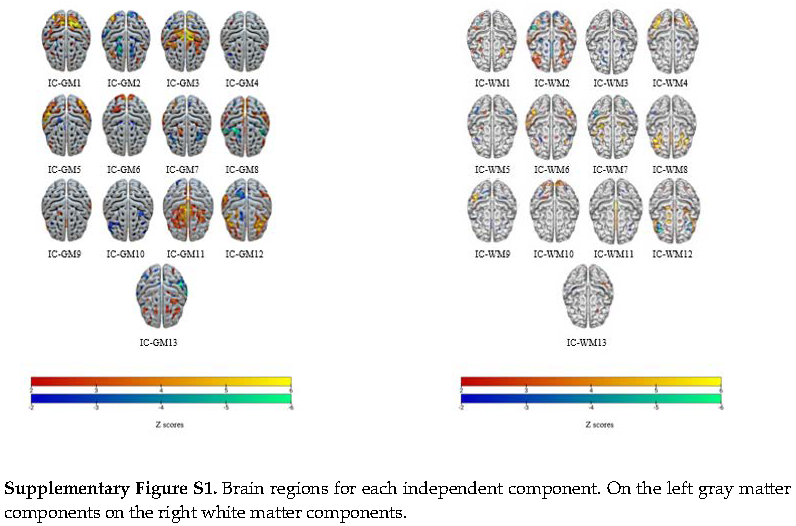

Supplement: Supplementary file 1 [file sensors-23-00610-s001.zip › Figure S1.png]
